# A Meta-Analysis on Mobile-Assisted Language Learning Applications: Benefits and Risks

**DOI:** 10.5334/pb.1146

**Published:** 2022-09-16

**Authors:** Mariela Mihaylova, Simon Gorin, Thomas P. Reber, Nicolas Rothen

**Affiliations:** 1 Faculty of Psychology, UniDistance Suisse, CH; 2 Department of Psychology and Educational Sciences, University of Geneva, CH

**Keywords:** memory, language acquisition, mobile-assisted language learning, language learning, meta-analysis, learning principles

## Abstract

Mobile language learning applications are a pervasive facet of modern life, however evidence on their effectiveness on L2 learning outcomes is lacking. In the current work, we sought to determine the effect of mobile language learning applications on L2 proficiency between groups who used mobile language learning applications and control groups who learned with traditional methods on L2 achievement. We systematically searched journal articles and grey literature between 2007–2019 and performed a quantitative meta-analysis based on 23 synthesized effect sizes. We also performed risk of bias and quality of evidence assessments on our included papers. We found a moderate-to-strong overall effect (*g* = 0.88) of learning achievement using mobile language applications compared to control groups who learned with traditional approaches. At the same time, we found high risk of bias and low quality of evidence across all included studies. Our results provide evidence for mobile applications as a beneficial tool for second language learning. However, findings should be treated with caution due to risks of high bias and low quality of evidence. Improvements for future studies are discussed.

## 1. Introduction

The last few decades witnessed an explosion of mobile application use for personal, professional and educational purposes. Mobile-learning refers to the use of mobile or portable devices such as smartphones or handhelds for learning (Viberg & Grönlund, 2012). Mobile-assisted language learning (MALL) refers to the use of mobile or portable devices for second language (L2) learning and encompasses a wide variety software such as using SMS to send/receive L2 vocabulary words (Kukulska-Hulme, 2012; Thornton & Houser, 2005; Viberg & Grönlund, 2012). MALL-application is a subset of MALL that refers to software on mobile devices *specifically* developed for the purpose of L2 learning (e.g., a mobile application developed specifically for vocabulary learning). Researchers and educators alike have recognized the potential benefits of MALL on L2 learning (Bano et al., 2018; Darmi & Albion, 2014; Kim & Kwon, 2012; Kukulska-Hulme, 2012; Viberg & Grönlund, 2012). However, despite the exponential growth and popularity of MALL, research on the efficacy of MALL-application for L2 learning is lacking. The primary goal of this work is to conduct a meta-analysis on the efficacy of exclusively MALL-applications on L2 achievement in comparison to traditional L2 learning approaches used in classroom settings (e.g., pen-and-paper approaches, textbook learning, taking notes, doing worksheets etc.).

MALL has been rapidly and readily applied in the educational context and is favored over other types of learning approaches. The various advantages of MALL, such as immediate access to learning material, portability, and personalization make them attractive tools for learning and may increase time spent learning (Kukulska-Hulme, 2012; Viberg & Gronlund, 2012). For example, research suggests that students learning a second language actively engage with MALL tools (Yaman, 2015) and 70% of students own a mobile phone and prefer to learn with mobile-learning approaches (Oz, 2013). Qualitative interviews and reports with students and teachers generally reflect positive experiences with MALL and its perceived effectiveness for language learning, increased learner satisfaction, increased motivation, increased motivation to learn on one’s own, and increased confidence (Ibrahim et al., 2017; Hwang et al., 2014; Lin et al., 2017; Kondo et al., 2012; Shadiev et al., 2018).

As the use of MALL in educational contexts increased, so did a market for MALL-applications, spurring the development of a multitude of MALL-applications created specifically for L2 learning. For example, mobile applications that support foreign vocabulary learning, such as mobile flashcards or mobile dictionaries, are now widespread (Bozdoğan, 2015; Fageeh, 2013; Mahdi, 2018; Rahimi & Miri, 2014; Thornton & Houser, 2005; Wu, 2014, 2015a, 2015b). A variety of MALL-applications have been designed and utilized to facilitate grammar learning and reading abilities (Ibrahim et al., 2017; Li & Hegelheimer, 2013; Ozer & Kılıç, 2018; Shadiev et al., 2018; Wu et al., 2012), writing skills (Hwang et al., 2014), as well as pronunciation and listening skills (Cavus & Ibrahim, 2017; Kondo et al., 2012). MALL-application systems that offer personalized content based on user learning levels, interest and learning cycles have also been developed (Chen & Chung, 2008; Chen & Hsu, 2008; Chen & Li, 2010; Hsu et al., 2013; Zou & Xie, 2018).

Crucially, most MALL-applications developed by industries are based on established learning principles from fundamental memory research. For instance, retrieval-based learning benefits learning over re-studying (Agarwal et al., 2021; Roediger & Butler, 2011; Yang et al., 2021), corrective feedback is more beneficial for learning than non-corrective feedback (Metcalfe, 2017), spaced learning is more effective than massed learning (Dempster, 1987; Kapler et al., 2015; McDaniel et al., 2013; Sobel et al., 2011) and multisensory encoding leads to more robust memory traces than unisensory encoding (Kast et al., 2011; Shams & Seitz, 2008). While it is possible to apply these learning principles in traditional learning contexts (e.g., retrieval-based learning with flashcards), these and other learning principles can be enforced by means of MALL-application (for a discussion of learning principles in information and communication technologies, see Reber & Rothen, 2018). Hence, it can be reasonably assumed that MALL-application learning is more efficient for L2 learning than traditional learning approaches.

Despite the diverse range of MALL-application utilized for educational purposes, their specific advantages over traditional approaches on L2 learning has not been systematically assessed in a meta-analytic approach. No prior meta-analytic work has focused exclusively on MALL-applications, rather assessing other general-purpose aspects of MALL (e.g., exercises sent via text messages or social-networking sites rather than only applications which are built for L2 learning). For instance, Sung and colleagues (2015) reviewed 44 journal papers and dissertations on MALL over 20 years (1993–2013) and found a medium overall effect of *d* = 0.55 in favor of L2 learning approaches with MALL tools in comparison to control groups who did not use the applications or used desktop computers. The authors also investigated the effects of different types of hardware (handheld devices, laptops, computers) and software (i.e., general purpose software and learning-oriented software) in their analysis, thus not concentrating solely on MALL-applications. Medium effects have also been reported in both Taj and colleagues (2016) and Cho and colleagues (2018) of *d* = 0.43 and *d* = 0.51 respectively for MALL on L2 language learning. These findings echo the results of other meta-analyses where medium effect sizes (*d* = 0.67) for vocabulary-learning with MALL compared to traditional-learning control groups were revealed (Mahdi, 2018). More recently, Chen and colleagues (2020) synthesized 80 experimental studies on MALL and found a medium-to-strong effect in favor of MALL over traditional-learning control groups. These studies all included different types of software, hardware and MALL tools (e.g. texting, gaming, social networking sites) as opposed to only mobile applications built specifically for L2 learning. Taken together, these data indicate compelling results in favor of adopting MALL for L2 learning. However, these data also demonstrate that the exclusive efficacy of MALL-*application* on L2 learning remains to be systematically investigated. Additionally, no previous work has considered both risk of bias and quality assessment of individual studies. These considerations are important to comply with current reporting standards and transparency for meta-analytic research (Maassen et al., 2020).

Therefore, a systematic literature search and quantitative meta-analysis performed in accordance with standard reporting guidelines is needed to better elucidate the effect of specifically MALL-application on L2 learning. The current work is, to our knowledge, the first meta-analysis to examine the effects of MALL-applications developed specifically for L2 learning and to assess the risk of bias and overall quality of the individual studies. Such an analysis is important to understand whether MALL-application can improve L2 language acquisition in comparison to traditional approaches. If this is the case, the analysis has the potential to further elucidate the most beneficial factors for L2 acquisition. We conducted a systematic meta-analysis using the Preferred Reporting Items for Systematic Reviews and Meta-Analyses (PRISMA: Page et al., 2021) to assess whether MALL-application compared to traditional learning approaches are more efficient when it comes to L2 acquisition. Furthermore, we explored which MALL-application factors are most beneficial for L2 acquisition.

## 2. Method

This meta-analysis followed the PRISMA (Page et al., 2021) guidelines for reporting. We ensured our meta-analyses abided by the transparency of data analysis and rigor of reporting as recommended by the field (for a review see Maassen et al., 2020). There is no protocol available for the current manuscript. All data and analysis have been made openly accessible on OSF (https://osf.io/htybd/).

### 2.1 Study Selection and Identification

#### 2.1.1 Systematic Literature Search

A systematic literature search was conducted on the MALL literature. The search strategy was similar to the method employed in Bano and colleagues (2018). The systematic literature search was completed in early-middle 2019 and the last date the reported databases were checked was December 2020. To retrieve sufficient and comprehensive literature, this study probed scientific articles published in peer reviewed journals from 2007–2019. We chose 2007 as the start date for our literature search as that was the year in which the first Apple iPhone was released, markedly changing the mobile and communication landscape thereon after. The databases used in our search were Springerlink, Ovid, ISI, Scopus and Learntechlib. The terms used to search the databases were: [language OR vocabulary OR lingu*] NEAR [learn* OR train* OR acquisition OR teach* OR lecture OR edu*] AND [mobile OR wireless OR seamless OR ubiquitous OR electronic OR digital OR smart] NEAR [learn* OR pedagog* OR device OR app* OR phone] AND [“non native” OR “non-native” OR second* OR foreign] WITH [tongue OR speech OR language]. The complete list of search strings can be found on OSF (https://osf.io/htybd/). An additional manual literature search was conducted on reference sections of prior meta-analyses and review papers published on MALL.

Grey literature was also probed to ensure we did not miss any relevant papers due to unpublished results, which could contribute to publication bias. We defined grey literature to extend to conference papers, dissertations, or other unpublished manuscripts on the field. The same search terms were used as our initial literature search. We also followed the same selection criteria as the general literature search, with the exception of the *“published in a peer reviewed journal”* criterion. We cross-referenced multiple pre-print archives on OSF (archives searched: OSF pre-prints, EdArXiv, MetaArXiv, Preprints.org, PsyArXiv) and unpublished dissertation repositories (Thesis Commons) in the education, psychology, and social sciences domains. One rater (MM) filtered through the searches for relevant titles and abstracts. In addition, we reached out to corresponding authors of included articles from our general literature search which met our inclusion criteria to inquire if they had any unpublished papers, null findings or any work in prep related to the topic. Authors were contacted by email and given 10 business days to respond to our request. They were informed that no answer by the end of the 10 business days meant a negative response from their part.

#### 2.1.2 Eligibility Criteria

In this meta-analysis, we were solely interested in MALL-application studies where a control group learned with traditional pen and paper methods and an experimental group utilized a mobile language learning application or mobile learning system only to learn a foreign language. This is critical to ensure we observe the difference in learning outcome between MALL-application use versus traditional classroom learning. Our second primary inclusion criterion pertains to the use of a mobile application or a mobile language learning system which served the exclusive purpose of L2 learning. By contrast, we did not consider studies on other mobile tools, which can be used for exercises around second language learning, but whose original purpose is entirely different (e.g., SMS, gaming, video recoding, electronic notepads). Other inclusion criteria included:

- Article is published in a peer-reviewed journal
- Article language is English
- Article is empirical, experimental or quasi-experimental
- Contains enough statistical information (means, standard deviations, and sample sizes of pre/post tests for experimental and control groups) to obtain an effect size
- L2 achievement is assessed as the main dependent variable in a post-test

#### 2.1.3 Study Screening

A research assistant (EB) screened the database created from our initial literature search for relevant titles and abstracts. Relevant titles were coded with a “1” or a “2” and irrelevant papers with a “0.” These titles were coded by three independent raters (EB, TR, NR) during the initial literature review stages. The abstracts and methods sections of papers coded with a “1” or “2” during screening were further read over and examined by MM. As a secondary step, key words (“mobile assisted language learning,” “effects of mobile language learning,” “language learning” and “vocabulary learning,” “personalized,” “learning system”) were applied on the database to ensure no relevant titles were missed.

### 2.2 Data Extraction and Bias Risk Assessment

Predetermined information (school level, learning focus, application name and type, duration of intervention, learning principle used, country of study origin) was extracted from each study and coded by three independent raters (MM, TR, NR). Risk of bias of individual studies included in our meta-analysis was assessed with Cochrane Risk of Bias 2 tool (Higgins et al., 2020; Sterne et al., 2019). Risk of bias is assessed across the following domains: the randomization process, deviations from intended interventions, missing outcome data, measurement of outcome, selection of the reported results. Judgments regarding the risk of bias for each domain are based on answers to signaling questions, which are rated on the basis of “yes,” “probably yes,” “no,” “probably no,” or “no information.” The resulting judgments of “low,” “some concerns,” or “high” risk of bias are outputted by the risk of bias algorithm in the tool. Two raters (MM and SG) served as independent raters for the risk of bias assessments.

### 2.3. Analyses

Statistical analyses were performed in R version 4.1.3 (*R Core Team*, 2020) using the packages designed for meta-analysis: ‘meta’ (Balduzzi et al., 2019, v5.2-0), ‘metafor’ (Viechtbauer, 2010, v3.0-2), ‘esc’ (Lüdecke, 2019, v0.5.1) and ‘dmetar’ (Harrer et al., 2019, v0.0.9000). To perform the meta-analysis, we followed the handbook guide by Harrer, Cuijpers, Furukawa, and Ebert (2021) titled *“Doing Meta-Analysis with R: A Hands-on Guide.”* All corresponding data and analysis, including raw data used to calculate effect sizes and analysis scripts, are available on OSF.

#### 2.3.1 Effect Size Calculation

Effect sizes were computed to represent the impact of MALL-application interventions on language learning for experimental groups who used the L2 mobile application or learning system versus control groups who used traditional pen-paper, classroom approaches. Effect sizes were all calculated in Hedges’ *g* (Hedges, 1981) using the R package ‘esc’ (Lüdecke, 2019). Studies containing more than one experimental group representing the same intervention category for the purposes of the review were pooled to create an overall intervention group and compared against the control to prevent unit-of-analysis error (Harrer et al., 2019; Higgins et al. 2019). Papers containing several outcomes for experimental and control groups, an effect size was calculated separately for each outcome, then averaged to obtain one overall effect size for that article. Effect sizes were interpreted based on the Cohen standard specifications (small = 0.2 and above, medium = 0.5 and above, large = 0.8 and above, Borenstein et al., 2009).

In case of missing or unclear information in the articles, we reached out to authors to obtain the required information. Authors were contacted by the e-mail address indicated as the corresponding author in the original paper. All authors contacted for additional information replied with the requested details.

#### 2.3.2 Small Study Effects and Publication Bias

Publication bias was assessed with different approaches. The first two encompass what is known as small study effects (SSE), which is the notion that small studies with large standard errors are most likely to generate non-significant findings because only very large effects in small studies would become significant and hence lead to publication bias (Harrer et al., 2021). As such, these approaches are referred to as “Small Study Effects” rather than publication bias (Schwarzer et al., 2015).

To assess SSEs, we first conducted Egger’s Test of the Intercept (Egger, 1997) which assesses the relationship between effect sizes and their corresponding standard errors. This relationship is illustrated with a funnel plot, and Egger’s Regression calculates whether asymmetry exists in funnel plot that could be due to publication bias. Because Egger’s Test is conducted on the effect sizes (i.e., standardized mean differences, SMDs), and the SMDs and standard error of included studies are independent, this process has been suggested to result in the inflation of false-positive results (Pustejovsky & Rodgers, 2019). To correct for this possibility, we conducted the Pustejovsky-Rodgers (2019). This approach uses a modified equation of the standard error when testing for funnel plot asymmetry which does not include the SMD itself, avoiding artificial correlation between the SMD and its standard error (Harrer et al., 2021).

To detect publication bias, we used three different quantitative methods recommended from the literature (Harrer et al., 2021). First, Duval and Tweedy’s trim-and-fill procedure (Duval & Tweedy, 2000) “trims” effect sizes with large standard errors from the funnel plot and “fills in” missing studies to maintain funnel plot symmetry. Second, we applied the PET-PEESE method (Stanley & Doucouliagos, 2014). In the PET method, the effect of small studies is controlled for by including the standard error as a predictor in a weighted regression model where the study’s effect size is regressed on its standard error (Harrer et al., 2021). Similarly, the PEESE method uses the squared standard error as a predictor. If the regression intercept calculated by PET is significantly larger than zero, the PEESE is used as the true effect estimate. If the PET intercept is not significantly larger than zero, the PET is used as the true effect estimate (Harrer et al., 2021). Lastly, we applied a selection model (McShane et al., 2016), which predicts how likely it is that a study is published (i.e., “selected”) based on its results (i.e., its *p*-value). We applied a three-parameter selection model, which is recommended if the number of studies is around 20 (Harrer et al., 2021). This model uses three parameters to assess publication bias: the effect size parameter, the heterogeneity parameter (tau^2^) and the likelihood of selection. The model then “removes” the assumed bias due to selected publication and derives a corrected estimate of the true effect (Harrer et al, 2021).

#### 2.3.3 Model Specification and Heterogeneity

We adopted a random-effects model approach to obtain an overall effect size measure based on the pooled weighted estimates from all the individual papers (Borenstein, 2009). The random-effects model assumes that the effects of individual studies deviate from the true intervention effect due to sampling variability and study variation because studies do not stem from the same population (Harrer et al., 2021). We used Knapp-Hartung adjustments (Knapp & Hartung, 2003) to calculate the confidence interval around the effect size, which is recommended to reduce false positives in case of a small number of studies (Harrer et al., 2021).

The random-effects model gives a measure of between-study heterogeneity, which is the extent to which effect sizes vary in a meta-analysis. Assessing heterogeneity is critical in a meta-analysis because there could be subgroups present in the data with a different true effect, or that there is no “real” effect behind the data meaning the studies included have nothing in common (Harrer et al., 2021). Under the random-effects model, heterogeneity is indicated by the *Q, I^2^, tau^2^* statistics. The *Q* statistic is the weighted sum of squared differences between individual effect size and the overall pooled effect across all studies and represents heterogeneity; *I^2^* is the percentage of variation in the effects that is not due to sampling error, or the degree of inconsistency in the meta-analysis; and *tau^2^* is the between-study variance in the meta-analysis and was calculated using the restricted maximum likelihood estimator (Viechtbauer, 2005) recommended for continuous outcome data (Harrer et al., 2021). Typically, an *I^2^* of 25% signals low heterogeneity, 50% signals medium, and 75% indicates substantial heterogeneity (Harrer et al., 2021), which is the rule of thumb we will adopt in the current analysis. The *Q* statistic is sensitive to both precision (the sample size of the study) and number of studies (*k), I^2^* is not sensitive to *k* but to precision, and *tau^2^* is not sensitive to either. Due to the limitations of each of these measures, it is not generally recommended to rely on just one but rather consider all. The prediction interval (*PI*) is defined as the range for which we can expect future studies to fall (Harrer et al., 2021) such that if the *PI* is positive in favor of the intervention, we can expect future studies would reflect this benefit in their effects. The *PI* is the recommended way to overcome the limitations with the heterogeneity statistics described above since it considers the between-study variance (Harrer et al., 2021). As such, we will use the *PI* as a proxy for the results we can expect for future MALL interventions.

#### 2.3.4 Outlier Analysis

To assess the robustness of our results, we conducted sensitivity analysis by investigating whether certain studies could be over-contributing to heterogeneity and therefore distorting our overall effect size. We tested for outliers using influential analysis in R (Harrer et al., 2021). Significant outliers are detected based on their respective weights on the pooled overall results and their contribution to the overall heterogeneity. The pooled overall effect was then recalculated with these outliers and influential cases removed and a corrected effect size is reported. These analyses were implemented using the ‘dmetar’ package in R (Harrer et al., 2019).

#### 2.3.5 Subgroup Analysis

We planned to look at subgroup effects by comparing the effects for 1) vocabulary and other types of language learning skills; 2) school level of participants (elementary, middle school, secondary school, university); 3) duration of intervention; 4) whether a pre-existing MALL-application or a language learning mobile application created by authors or researchers was used in the intervention; 4) learning principles (described in section 2.3.6). Assessments for subgroup inclusion were based on inclusion criteria (see Supplementary Materials, Criteria for Subgroup Analysis) which each rater was equipped with during the rating sessions. If the necessary information for subgroup classification could not be located in the respective article during rater deliberation sessions, the paper was excluded from classification and removed from further subgroup analysis. In case of disagreement, the three raters went through several rounds of deliberations to discuss differences in classifications until consensus was reached. Inter-rater reliability was assessed with Fleiss’ Kappa scores before rater deliberation (see Supplementary Materials, Table S2). This deliberation process was identical for the learning principles exploratory analysis (section 2.3.6). Because some articles contained a mix of language learning skills such as listening and vocabulary (*N* = 1) or grammar, writing and reading (*N* = 1), these were combined into one category to denote mixed language learning skills. Subgroup analysis was performed by calculating the random-effects model to test for between subgroup differences using the ‘dmetar’ package in R (Harrer et al., 2019).

#### 2.3.6 Learning Principles: Exploratory Analysis

We were interested to examine whether learning principles could be used to better understand, and potentially predict, the effectiveness of learning using mobile applications. We classified the intervention of each article based on whether learning principles were employed in the MALL-application. The learning principles included feedback, retrieval, distributed learning, and multisensory learning, all of which have been identified by memory research to be beneficial for learning (Reber & Rothen, 2018; Weinstein et al., 2018).

The same three raters (MM, TR, NR) rated each paper across the four principles based on inclusion criteria (see Supplementary Materials, Table S1). Effect sizes were computed for each learning principle by pooling all papers which were coded as having included a particular learning principle and compared with the studies that did not include the learning principle in question.

### 2.4. Quality of Evidence

Quality of evidence was assessed in this meta-analysis with the Grading Recommendations Assessment, Development and Evaluation (GRADE; Guyatt et al., 2008) using the GRADEpro Guideline Development Tool available online (gdt.gradepro.org). Quality of evidence is assessed across five domains: risk of bias, inconsistency of trial results, indirectness of measure, imprecision of effect size estimate, and possible publication bias. Judgments across the domains are rated as “not serious,” “serious,” and “very serious” based on the likelihood of studies to be upgraded or downgraded for quality on each criterion.

## 3. Results

### 3.1 Systematic Literature Search

**Figure 1:**
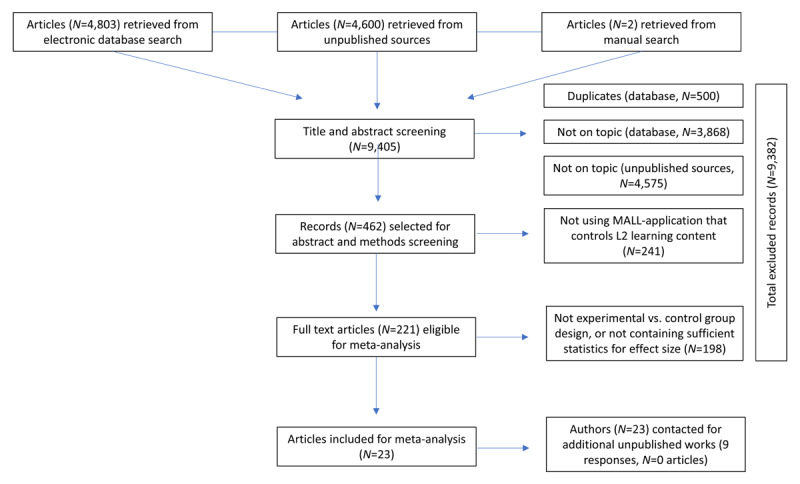
Flow chart of the literature search process.

Figure 1 depicts the PRISMA flowchart of the literature review and screening process. We located a total of 4,803 research articles. After deletion of doubles, 4,303 articles remained, establishing our initial literature database for screening. Following article screening and abstract and method check, we were left with 18 papers that fit all inclusion parameters. Two additional papers had already been identified through references check were integrated for a total of 20 articles that fit all inclusion criteria. Articles which initially fit our inclusion criteria were excluded upon further inspection due to either utilizing other features of MALL (i.e., movie-maker or notepads) instead of MALL-application specific for L2 learning (Khodabandeh & Soleimani, 2017; Li & Tong, 2019) or due to lack of control group (Chen & Chung, 2008; Chen & Hsu, 2008; Li & Hegelheimer, 2013; Zou & Zie, 2018).

Probing the grey literature search yielded 4,600 unpublished studies from pre-print archive servers. Twenty-five titles were identified as potentially relevant, however after more thorough examination of the methods section, no papers met our inclusion criteria for grey literature. We additionally scanned our initial literature search database (4,303 titles) and references checks for any relevant unpublished articles or conference papers. This yielded three results. The unpublished conference papers were integrated in our overall batch for a total of 23 articles for quantitative analysis. A total of 9 authors out of the 25 contacted responded to our inquiry regarding unpublished work or null findings, however all the respondents confirmed they did not have any such data to share. Thus, our total articles for inclusion were 23.

#### 3.1.1. Characteristics of Included Studies

Descriptive information for all studies included in this meta-analysis for statistical analysis is presented in Table 1. All included studies are preceded by a code beginning with letter ‘A’ and will be referred to with this code for the rest of the work. A total of 23 articles total were identified for quantitative synthesis, with a sample size of 963 participants in the experimental group and 910 participants in the control group (total *N* = 1,873). Of these 23 articles, 20 were published in scientific journals and three were retrieved from conference papers (i.e., grey literature).

**Table 1:** Table of articles included in this meta-analysis.


CODE	AUTHOR	TOTAL N	AGE	DURATION	FOCUS	TARGET LANGUAGE	APPLICATION	DESIGN	ORIGIN COUNTRY	TYPE	SOURCE

A1	Lan & Lin, 2016	34	17–23	4 weeks	Communication	Chinese	Mobile Seamless System (MOSE)	Between-subjects, Mixed Methods	Taiwan	Journal	jstor.org/stable/10.2307/jeductechsoci.19.3.335

A2	Rachels & Rockinson-Szapkiw, 2017	167	3^rd^and 4^th^graders	12 weeks	Vocabulary & Grammar	Spanish	Duolingo	Quasi-Experimental	United States	Journal	https://doi.org/10.1080/09588221.2017.1382536

A3	Sandberg, Maris, & Geus, 2011	75	8–10	1 day	Vocabulary	English	MEL Application	Quasi-Experimental	Netherlands	Journal	https://doi.org/10.1016/j.compedu.2011.01.015

A4	Basoglu & Akdemir, 2010	60	17–24	6 weeks	Vocabulary	English	ETACO mobile flashcards	Between-Subject, Mixed Methods	Turkey	Journal	ERIC Number: EJ898010 https://eric.ed.gov/?id=EJ898010

A5	Cavus & Ibrahim, 2017	37	10–13	4 weeks	Listening, Vocabulary, Comprehension Pronunciation	English	Near East University Children’s Story Teller (NEUCST)	Between-Subjects	Turkey	Journal	https://doi.org/10.1111/bjet.12427

A6	Azabdaftari & Mozaheb, 2012	80	21	7 weeks	Vocabulary	English	Spaced Repetition System (SRS)	Between-Subject, Mixed Methods	Iran	Journal	ERIC Number: EJ1064983 https://eric.ed.gov/?id=EJ1064983

A7	Ibrahim, Chee & Yahaya, 2017	53	Primary school	4 weeks	Reading	Chinese	Learn Chinese Mandarin App	Quasi-Experimental	Malaysia	Journal	https://doi.org/10.1504/IJMLO.2017.10005992

A8	Ozer & Kilic, 2018	63	18–22	6 weeks	Grammar	English	Variety of mobile apps	Not Explicit, Between-Subjects	Turkey	Journal	https://doi.org/10.29333/ejmste/90992

A9	Kilickaya & Krajka, 2010	38	17–19	5 weeks	Vocabulary	English	WordChamp	Between-Subjects	Turkey	Journal	ERIC Number: EJ898003 https://eric.ed.gov/?id=EJ898003

A10	Wu, 2014	50	20–23	1 semester	Vocabulary	English	Word Learning	Not Explicit, Between-Subjects	China	Grey	https://doi.org/10.1016/j.sbspro.2014.07.409

A11	Wu, 2015a	70	20–23	8 weeks	Vocabulary	English	Word Learning-CET6	Not Explicit, Between-Subjects	China	Journal	https://doi.org/10.1016/j.compedu.2015.02.013

A12	Wu, 2015b	199	20–22	1 semester	Vocabulary	English	Word Learning-CET4	Not Explicit, Between-Subjects	China	Journal	https://doi.org/10.1371/journal.pone.0128762

A13	Fageeh, 2013	58	University students	1 semester	Vocabulary	English	Android Online Dictionary	Not Explicit, Between-Subjects	Saudi Arabia	Journal	web.a.ebscohost.com

A14	Rahimi & Miri, 2014	34	University students	16 sessions	Vocabulary	English	Longman Mobile Dictionary	Not Explicit, Between-Subjects	Iran	Grey	https://doi.org/10.1016/j.sbspro.2014.03.567

A15	Kondo et al., 2012	88	University students	April – July semester	Listening & Reading	English	TOEIC	Not Explicit, Between-Subjects, Mixed Methods	Japan	Journal	https://doi.org/10.1017/S0958344012000055,

A16	Wang, 2016	196	University students	1 semester	Reading	English	Learn English Audio & Video	Between-Subjects	Taiwan	Journal	https://doi.org/10.1080/10494820.2015.1131170

A17	Wang & Shih, 2015	93	19	1 semester	Vocabulary	English	The Most Important 2000 TOEIC Words	Not Explicit, Between-Subjects	Taiwan	Journal	https://doi.org/10.1504/IJMC.2015.070060

A28	Hwang, Chen, Shadiev, Huang & Chen, 2014	59	6^th^grade	3 classes per week for 1.5 months	Writing	English	Situated Writing System	Quasi-Experimental	Taiwan	Journal	https://doi.org/10.1080/09588221.2012.733711

A19	Shadiev, Hwang, Liu, 2018	53	13–14	3 weeks	Grammar, Writing, Reading	English	Mobile Multimedia Learning System (MMLS)	Quasi-Experimental	Taiwan	Journal	https://doi.org/10.1007/s11423-018-9590-1

A20	Wu, Sung, Huang, Yang, Yang, 2011	113	University students	7 weeks	Reading	English	Ubiquitous English-Reading Learning System	Not Explicit, Between-Subjects	Taiwan	Journal	www.jstor.org/stable/10.2307/jeductechsoci.14.4.164

A21	Hsieh, Wang, Su, Lee, 2012	60	University students	4 months	Reading & Vocabulary	English	Fuzzy, Logic-Based Personalized Learning System	Between-Subjects	Taiwan	Journal	www.jstor.org/stable/10.2307/jeductechsoci.15.1.273

A22	Zhang, 2016	120	University students	10 weeks	Listening Comprehension	English	Keke English & Easy IELTS	Between-Subjects	China	Grey	https://doi.org/10.12783/dtssehs/icaem2016/4290

A23	Lee, 2014	120	15–21	20 classes	Vocabulary	English	Teacher-Created Application	Not Explicit, Between-Subjects	Taiwan	Grey	https://doi.org/10.1109/MCSoC.2014.24


Over half (65%, *N* = 15) of included papers were conducted in Asia, with Taiwan (*N* = 8) and China (*N* = 4) being the most common areas, followed by Malaysia (*N* = 1) and Japan (*N* = 1). The second most common geographic area was the Middle East (26%) comprising of Turkey (*N* = 4), Iran (*N* = 1), and Saudi Arabia (*N* = 1). Finally, Western countries contributed to only 8% of included studies with one from the USA (A2) and only one from Europe (Netherlands, A3). The target language to be learned was English in nearly all papers, with one paper learning Spanish (A4) and two Chinese Mandarin (A1, A7). All studies were published after 2010.

The majority of papers (70%, *N* = 16) were conducted on university aged students. The remaining 30% of included studies were conducted on younger children at the elementary school level (until 6^th^ grade; *N* = 3) and middle school aged children (6–9^th^; *N* = 2), and high school (13–14+ years, *N* = 2). In one instance (A14), there was unclear information for participant ages. The MALL-application intervention durations varied greatly – with the shortest intervention comprising of 1 day (A5) and the longest duration was 4 months (A21). The most frequent interventions were between 2–6 weeks (*N* = 7) and an entire semester (*N* = 7).

Mobile language learning applications were used in 18 studies, while the rest (*N* = 5) employed a mobile language learning system approach which also ran as a mobile application. A considerable amount (43%, *N* = 10) of all applications used were created or designed by the authors and the other 13 papers featured already pre-existing mobile applications (57%). Roughly half of papers (47%, *N* = 11) targeted vocabulary learning, while the second major learning focus was reading (13%, *N* = 3). Grammar, listening comprehension, and writing followed, with each focus being about 4% each. The remaining studies featured a combination of language learning aspects such as communication (*N* = 1); vocabulary, pronunciation, listening, comprehension (*N* = 1); vocabulary & grammar (*N* = 1); listening and reading (*N* = 1); reading and vocabulary (*N* = 1); and grammar, writing and reading (*N* = 1).

All studies featured a between-subject experimental or quasi-experimental design. Only 5 studies (22%) explicitly stipulated that participants were randomized in experimental and control groups. The remaining 78% of studies (*N* = 18) reported convenience or purposeful sampling (*N* = 4), or unclear sampling and randomization methods (*N* = 14). In these studies, participants were frequently allocated to the experimental or control group based on what classroom they were in, whether their mobile phone was compatible with the MALL-application to be utilized in the study, and whether they wanted to use a mobile device or work with traditional approaches.

All studies featured a pre-test and post-test design. Vast differences in L2 measurements were seen across all studies, with each paper utilizing a different language test to measure performance (i.e., no article utilized the same language learning measurement tool). The measurement scales were either instructor-created or an academic test.

Out of the 23 included papers, only three (13%) reported follow up results to assess language learning after the primary intervention had taken place. In Kilickaya and Krajka (2010), a follow-up post-test measure was conducted three months after the main intervention to assess performance between the experimental and control group once more. Lee (2014) reported delayed post-test results for experimental and control groups taken one week after the intervention. Lastly, Kondo and colleagues (2012) conducted a second experiment where 15 out of the 42 original participants from the experimental group in their first study assessing the MALL-application intervention were recruited to determine whether students continued using the mobile application. With no control group involved, we thus did not calculate an effect size for the follow-up experiment.

#### 3.1.2. Risk of Bias

**Figure 2:**
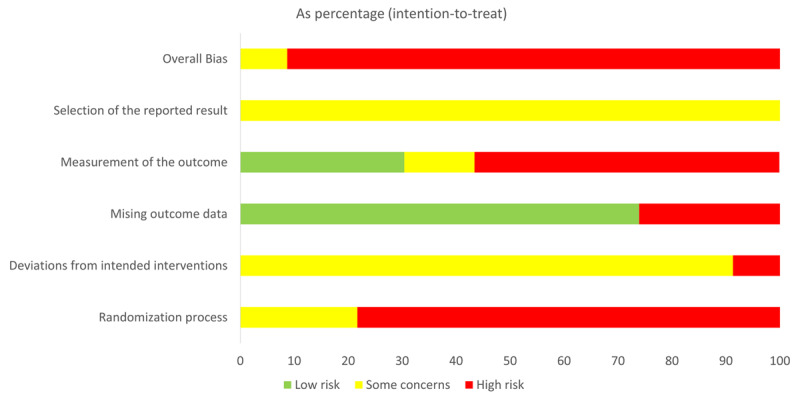
Risk of bias across studies. *Note*: Summary table of the risk of bias in all included studies overall and across each of the five domains: overall bias (high risk), selection of the reported result (some concerns), measurement of the outcome (a mix of low bias and some concerns, but mostly high risk), missing outcome data (predominantly low risk), deviations from intended interventions (largely some concerns), randomization process (mostly high risk). The bias domain is seen on the *y*-axis, and the score out of 100 is illustrated on the *x*-axis.

Risk of Bias was assessed with the Cochrane RoB 2 tool (Higgins et al., 2020; Sterne et al., 2019; see Figure 2). The total risk of bias was “high” across all studies overall. No study featured true random allocation (i.e., random number generator or table) of participants into experimental or control groups, opting for convenience sampling, no information, or merely stating that randomization occurred. The majority of papers (91.3%) posed as “some concerns” for deviations from intended intervention, due to all participants who were recruited for the intervention or control groups be analysed as such (i.e., no participant switched groups during the intervention) and due to researchers being aware of what intervention was administered to which group of students (i.e., not blinded). Most papers (73.9%) did not include enough information on missing outcome data. If missing outcome data was reported, there was usually poor justification for why it was removed, and no sensitivity analyses were done on the data to test how the removed data points impacted overall results. In terms of measurement of the outcome, over half (56.5%) of articles did not describe what the language measurements entailed during the post test, mentioning only that a post-test was carried out. Overall, there was little rationale explaining how or why the chosen pre- or post-test questions were chosen to measure the particular language learning facet that they were measuring, nor was there any reliability measures done on these tests in most cases. However, the measurement of the outcome was comparable across both experimental and control groups at all time points measured (i.e., all participants received a pre- and post-test at the same time). No study referred to a study protocol established prior to the intervention, although several (*N* = 3) stipulated an analysis plan prior to presentation of experimental results. Two (MM and SG) completed RoB ratings independently and then discussed discrepancies until a consensus was reached.

### 3.2. Meta-Analytic Results

#### 3.2.1. Publication Bias

**Figure 3:**
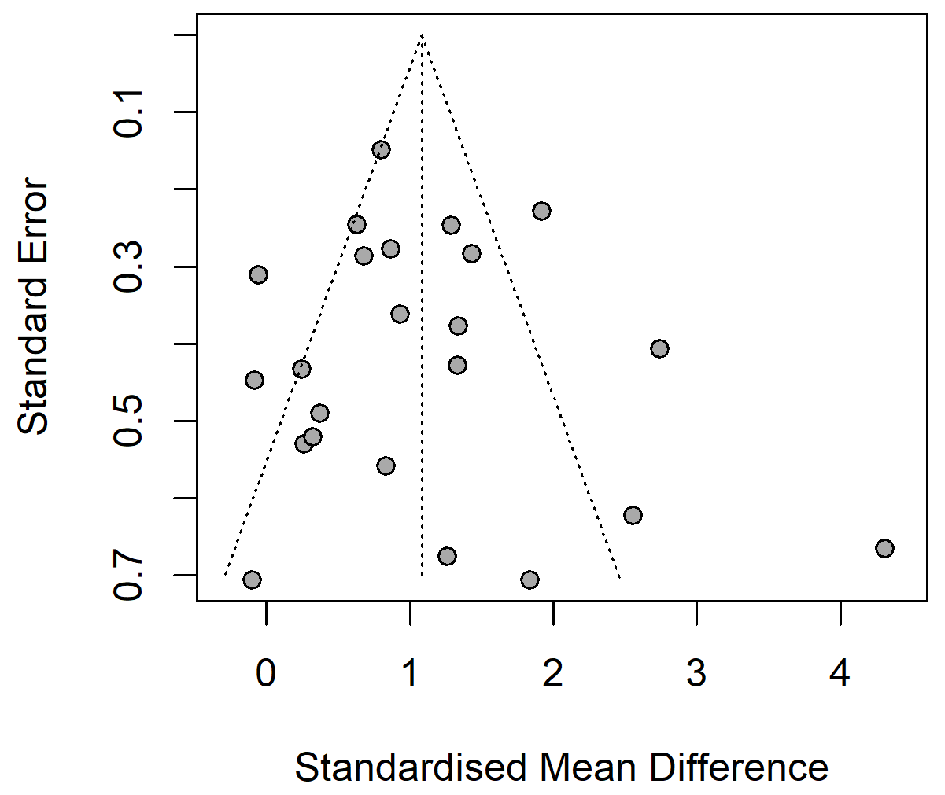
Funnel plot of all included studies.

Publication bias was assessed in this meta-analysis with several methods. We first report the results of the SSE tests. Egger’s test of the intercept (Egger et al., 1997) to quantify the potential asymmetry in the funnel plot and test for significance. The result of Egger’s test indicates no significant asymmetry present (*p* = 0.58), suggesting no significant publication bias is present in the current sample (Figure 3). Additionally, the Pustejovsky-Rodgers (2019) correction revealed no funnel plot asymmetry (*p* = 0.23), mirroring the results of Egger’s Test. Taken together, these measures suggest no funnel plot asymmetry present in our analysis and thus no publication bias due to small study effects.

Additional measures of publication bias we conducted were the trim-fill procedure (Duval & Tweedy, 2000), the PET-PEESE approach (Stanley & Doucouliagos, 2014) and the three-parameter selection model (McShane, 2016). Results of the trim-fill procedure showed that the estimated number of missing effects was zero with zero additional studies being “filled” in, suggesting little risk for publication bias. Because the PET regression intercept was not significantly greater than zero (*g* = –0.036, *p* > 0.96), this suggests little publication bias. The results of the three-step parameter selection model results revealed no significant evidence of publication bias (*LRT =* 0.0015, *p* = 0.97). Moreover, the true effect size estimate of 1.07 (*95% CI*: 0.50–1.65), which is nearly identical to our overall pooled estimate under the random-effects model (*g* = 1.08). This indicates our meta-analysis was not biased by a lower selection probability of non-significant results.

#### 3.2.2 Overall Effects for MALL-application interventions

**Figure 4:**
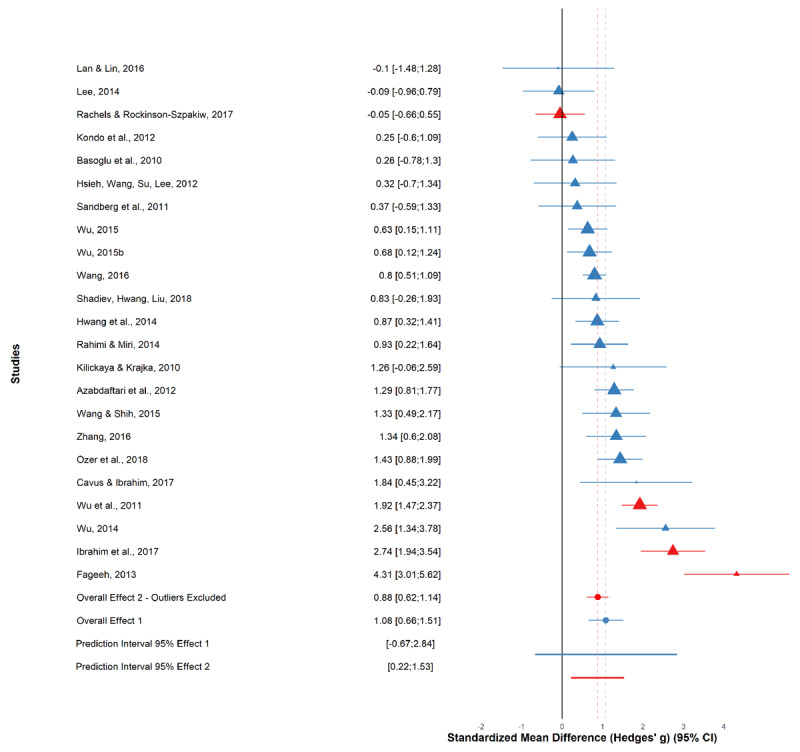
Forest plot of all studies and overall effects. *Note*: Forest plot of all studies included in the meta-analysis. Individual effect sizes of all studies included in this meta-analysis with their respective weight in the analysis and confidence intervals represented by the triangles. Larger triangles indicate more weight in the random effects model. Red triangles denote the outliers identified in the analysis. The *x*-axis represents the standardized mean difference effect size (Hedges’ *g*) and the *y*-axis is each individual study with its 95% confidence interval. The overall effect sizes are represented by the red and blue circles at the bottom. The 95% *PI* denotes the 95% prediction intervals for the overall effect of all studies (blue) and for the overall effect with outliers removed (red), indexed by the thick red and blue lines.

A total of 23 effect sizes were computed in this meta-analysis and pooled under the random-effects model with Knapp-Hartung adjustment (2003) to obtain the overall effect of MALL-application on L2 learning achievement (Borenstein et al., 2009) between experimental groups who utilized the MALL-application and control groups which used traditional pen-paper approaches. Overall, there is a large effect of *g* = 1.08 (*CI* 95%: 0.66–1.51, Figure 4) under the random effects model suggesting that MALL-application use facilitates L2 learning in the treatment group who used the application in question compared to the control group who learned via traditional approaches. The *Q* statistic is significant and suggests the overall sample is highly heterogenous (*Q* = 106.10 *p* < 0.001, *df* = 22). This further supported by the *I^2^* statistic of 79%, suggesting a 79% chance of results being due to heterogeneity rather than chance. A medium-to-large *tau^2^* of 0.67 indicates medium-to-high between-study variance. As each measure of heterogeneity is limited due to sensitivity to statistical power and precision, we additionally consider the prediction interval for a more robust estimate (Harrer et al., 2021). The prediction interval (*95% PI*: –0.67 to 2.84) falls slightly below zero, meaning we cannot be entirely certain that the strong positive effect we observe is robust in every sense.

#### 3.2.3 Outlier Analysis

To further investigate the contributions to significant heterogeneity (*Q* = 106.10, *p* < 0.001; *I^2^* = 79%) in our sample, we ran outlier and influence analysis to detect and eliminate potential outliers in our sample that could be contributing to the between-study variance. A total of four influential cases (A2, A7, A13, A20; see Supplementary Materials, Figure S1) were detected out of our total 23 studies.

A new overall effect size is recalculated with the weights of the four identified outliers set to zero in the random-effects model. The new overall effect size now stands at lower, but still strong *g* = 0.88 (*95% CI*: 0.62–1.14, *95% PI*: 0.22–1.53; see Figure 4). The *Q* statistic is still significant (*Q* = 32.72, *df* = 18, *p* = 0.02), however markedly reduced indicating a substantial reduction in heterogeneity. Further, *I^2^* (45%) suggests a low to medium chance of the results being due to heterogeneity rather than chance and a minimal *tau^2^* (0.083) suggests virtually non-existent between-study variance. The greatly reduced *tau^2^* value after outlier removal additionally suggests that a great deal of the heterogeneity was caused by outliers. The positive prediction interval (95% *PI*: 0.22–1.53) suggests that we can be relatively confident that future studies would find a similar effect (Harrer et al., 2021), a confidence we did not observe with the prediction interval of the initial model (*g* = 1.08, *95% PI*: –0.67–2.84). Overall, results with outliers removed suggest a decreased, yet still moderate-to-strong, effect size with low heterogeneity and a positive prediction interval.

#### 3.2.4 Subgroup Analysis

We conducted subgroup analysis for school level of learners (elementary school, middle school, secondary or high school, university) learning focus (vocabulary, reading, writing, grammar, or a mix of language learning skills), duration of reported intervention, type of application used (pre-existing or developed by authors) and learning principles. Analyses were conducted with the four outliers (see *section 3.2.3*) removed (Table 2). Medium-to-strong effects were seen across all subgroups, indicating a beneficial impact of MALL-application across the board. No significant differences were observed between any subgroups. Inter-rater reliability scores for all subgroups were above 0.70, indicating good inter-rater agreement (see Supplementary Materials, Table S2).

**Table 2:** Summary of findings table.


*INTERVENTION (MALL-APPLICATION VS. CONTROL)*	*N*	*G*	*95% CI*	*TAU^2^*	*I^2^*	*Q*	*95% CI*

Overall	23	1.08	0.66–1.51	0.67	79.3%	106.10***	<0.0001

Overall, outliers removed	19	0.88	0.62–1.14	0.08	45%	32.72*	0.018

***INTERVENTION (MALL-APPLICATION VS. CONTROL)***	***N***	***G***	***95% CI***			***Q_BETWEEN-GROUPS_***	***P-VALUE***

*School Level*							

University	14	0.87	0.51–1.23	0.16	56.3%		

High School	1	0.83	0.26–1.93	*NA*	*NA*	1.44	0.84

Middle School	2	1.13	–4.39–6.66	0.18	39.1%		

Elementary School	1	0.37	–0.59–1.33	*NA*	*NA*		

*Learning focus*							

Vocabulary	10	0.87	0.41–1.33	0.17	55.1%		

Reading	1	0.80	0.51–1.09	*NA*	*NA*		

Writing	1	0.87	0.32–1.41	*NA*	*NA*	7.99	0.24

Grammar	1	1.43	0.88–1.99	*NA*	*NA*		

Communication	1	–0.10	–1.48–1.28	*NA*	*NA*		

Listening	1	1.34	0.60–2.08	*NA*	*NA*		

Mix of language skills	4	0.65	–0.38–1.67	0.06	28.6%		

*Intervention Duration*							

1 day	1	0.37	–0.59–1.33	*NA*	*NA*		

2–6 weeks	6	0.98	0.27–1.70	0.17	38.6%		

7–10 weeks	4	0.99	0.45–1.54	0.05	35.6%	2.34	0.67

1 semester	6	0.89	0.14–1.65	0.23	58.9%		

10–16 sessions (undefined weeks)	2	0.46	–5.97–6.89	0.35	67.8%		

*App Type*							

Pre-existing App	10	0.98	0.68–1.28	0.05	33.8%	0.63	0.43

Developed App	9	0.76	0.20–1.32	0.19	52.5%		

*Learning Principles*							

Retrieval Practice	12	0.95	0.56–1.34	0.13	51.1%	2.57	0.28

No-Retrieval Practice	6	0.73	0.35–1.11	0.006	28.1%		

Feedback	9	0.89	0.58–1.20	0.02	26.6%	0.01	0.91

No-Feedback	10	0.86	0.37–1.35	0.22	58.7%		

Multimodal	14	0.87	0.53–1.22	0.12	51.8%	0.03	0.87

No-Multimodal	5	0.91	0.33–1.49	0.07	23.1%		


*Note*: “*” = *p* < 0.01, “**” = *p* < 0.001, “***” = *p* < 0.0001. Q_between-groups_ is the denotes the *Q* statistic for between-groups comparison, *NA* denotes unavailable values due to small number of studies.

#### 3.2.5 Follow up effects

Follow-up measurements between experimental and control groups were conducted in only two studies. In Kilickaya & Krajka (2010), the effect size for the follow-up post test conducted 3 months after the intervention was of *g* = 0.20 (*95% CI*: –1.07–1.48), indicating a small effect of continued learning three months post MALL-application intervention between the experimental and control groups. Lee (2014) also reported a delayed post-test one week after the MALL-application intervention characterized by a small effect size (*g* = 0.13; *95% CI*: –0.75–1.00). Overall, these results show a weak effect of MALL-application versus control group at delayed post-test, suggesting some sustained effects of MALL-application benefit after a delay, although there is not enough evidence currently to say for certain.

### 3.3 Exploratory Analysis: Learning Principles

We explored whether MALL-applications contained elements of learning principles (retrieval practice, feedback, distributed learning, multisensory learning) in the way they controlled learning content. Overall, all applications or learning system included some type of learning principles. Distributed learning was found present in every paper and was thus excluded from further statistical analysis. Strong effect sizes were also observed for all learning principles (see Table 2). The second most common learning principle was multimodal learning (*g* = 0.87, *N* = 14), followed by retrieval practice (*g* = 0.95, *N* = 12), and feedback (*g* = 0.89, *N* = 9). No significant differences between were observed between using a learning principle versus no principles used. Combinations of learning principles in one article were not investigated due low number of articles.

### 3.4 Quality of Evidence

To ascertain the quality of evidence in our included studies, we performed GRADE assessments across the domains of our subgroups: school level, learning focus, intervention duration, learning app type (available on OSF). Outcomes were assessed across the five GRADE domains: risk of bias, inconsistency, indirectness of evidence and imprecision. The overall quality of evidence was poor, with “low” and “very low” being the most frequent GRADE assessment awarded for each outcome. The primary reasons for low certainty assessments were: a) high risk of bias across papers, b) high degree of inconsistency of results as indicated by *I^2^* estimates of over 40% in some cases, c) relatively imprecise measures as indicated by wide confidence intervals around each effect size for each subgroup. Indirectness of evidence was sometimes judged as “not serious” due to measurements being directly related to the outcome of interest (i.e., target word exams as a direct measure of vocabulary learning). Inconsistency was judged as “not serious” in some cases, as indexed by low *I^2^* values in some subgroups. Taken together, this analysis suggests that future studies might impact the overall effects and their confidence intervals found in the different outcomes assessed observed in the current work.

## 4. Discussion

This meta-analysis examined the effects of utilizing MALL-application in experimental groups over control groups who used traditional pen-paper classroom methods on L2 learning achievement. Our analysis revealed a strong overall effect in favor of MALL-application (*g* = 1.08, *N* = 23) over traditional-approach control groups on L2 learning. After outlier exclusion, we observed a moderate-to-strong effect (*g* = 0.88, *N* = 19). Publication bias was not detected in our sample. Subgroup analysis revealed moderate-to-strong effects across all moderator variables. In terms of overall effects sizes, our results seem to offer positive effectiveness for MALL-applications on L2 acquisition, however these results should be approached with caution as our results also revealed high risk of bias and overall low quality of evidence across all articles and outcomes in the meta-analysis.

Nearly all articles in our meta-analysis revealed positive effect sizes in favor of MALL-application in comparison to traditional approaches. These findings echo results from previous meta-analyses that report positive medium-sized effects for L2 learning using MALL more generally (Chen et al., 2020; Cho et al., 2018; Mahdi, 2018; Sung et al., 2015, 2016; Taj et al., 2016). That is, the current finding extends beyond prior literature because it elucidates that MALL-*application* specifically provides a benefit over traditional learning approaches, as opposed to general MALL technology on learning previously conducted. Moreover, the observed moderate-to-strong in comparison to previously found moderate effects suggests that MALL-application might be slightly more beneficial than the more general MALL approach.

Both SSE and publication bias measures detected little to no publication bias in the current sample. This finding corroborates previous literature as far as SSE are concerned (e.g., Cho et al., 2018). However, our analysis went a step further by analyzing publication bias with multiple approaches not included in prior reviews on the MALL topic. It is important to note that the results of the SSE and publication bias approaches described in section 3.2.1 are with their limitations and weaknesses. For example, the PET-PEESE is prone to over-adjusting effects and leading to underestimation of the true effect size (Carter et al., 2019) and the trim-fill method is known to under-correct for publication bias (Harrer et al., 2021). Additionally, both the trim-fill and PET-PEESE methods are not robust when the heterogeneity is high, as is the case here. The three-parameter selection mode has been found to be more reliable than other methods (McShane et al., 2016), however it can be difficult to interpret (Harrer et al., 2021). Moreover, it is important to note that publication bias can be caused by a multitude of other factors, such as between-study heterogeneity and high risk of bias within studies (Harrer et al., 2021). Thus, although we found no quantifiable publication bias, the high risk of bias (see section 3.1.2) may be an alternate explanation for potential publication bias.

Only 23 studies fulfilled the inclusion criteria of our meta-analysis on MALL-application, which is surprising given the popularity of mobile devices and learning applications used today. One explanation for this low sample size is that a plethora of MALL-applications exist and continue to be utilized in educational contexts or for individual L2 learning, but remain experimentally unvalidated for learning outcomes. That is, students and teachers may be using various MALL-applications without awareness as to their actual effects on learning. This resounds Burston’s (2015) review, where it is found that over 40% of all articles published on MALL are unrelated to MALL-applications more specifically and an overwhelming majority of articles lack quantifiable learning outcomes. Given our finding that MALL-application might be more effective than general MALL on L2 learning, these findings thus highlight the importance of using MALL applications that are both specifically designed for learning *and* have been experimentally validated for learning outcomes. Translating this to practical terms, it is advisable for educators and students to be sparing in terms of the applications they utilize in the classroom and limit their use to only validated MALL-applications. At the same time, pre-existing or newly developed MALL and MALL-applications should be experimentally validated to ensure quantifiable learning outcomes prior to use in classrooms or on the market for individual use.

It has long been deemed necessary to integrate learning principles from fundamental memory research within mobile learning tools to enhance learning (Parsons & Ryu, 2006; Reber & Rothen, 2018; Zydney & Warner, 2016). We attempted to address this need by exploring whether the four learning principles of retrieval practice, feedback, distributed learning, and multimodal learning are utilized in MALL-application to boost L2 learning. Our analysis revealed that all MALL-applications used in all included articles featured distributed learning and included one or more learning principles. However, due to the small number of studies in the different subgroups, we were not able to identify differential effects. Critically, none of the learning principles were directly manipulated by authors in the studies. It will thus be an interesting endeavour for future studies to incorporate and manipulate learning principles to directly assess the relative contribution of each principle on learning and their interactions on L2 learning with a MALL-application. Such studies would be interesting in their own rights for the field of memory research more generally because our current knowledge on the interaction of different learning principles is extremely limited (Belardi et al., 2021; Weinstein et al., 2018). Given how easy it is to collect large amounts of data across extended time periods with MALL-application, such studies therefore offer a unique opportunity to advance current knowledge in the field of basic memory research beyond mere L2 learning.

Only three studies in our included batch administered a delayed post-test following the intervention, with follow-up effects. This finding suggests some sustained positive benefit on L2 learning with MALL-application over the long-term, however given the small number of studies it is premature to draw conclusion on potential long-term benefit. Elucidating the long-term value of MALL-application beyond the intervention period requires a data-driven approach as some students might continue to use the MALL-application beyond the experimental intervention period (Kondo et al., 2012; Sandberg et al., 2011). In addition, because most studies investigated only short intervention periods, it might be the case that the effects of MALL-application interventions diminish over the time (Sung et al., 2016).

Besides the overall finding that MALL-application is likely to be beneficial for L2 learning achievement, we also identified several potential risks. Due to the applied nature of the research, most studies lacked proper randomization procedures in assigning participants to experimental and control groups (i.e., risk of bias). Additionally, articles lacked transparency when it came to how outcome variables were assessed. It is thus recommended for future articles on MALL-application interventions to provide scoring or coding schemes for how post-test questions were graded, as well as justification for why certain target words were chosen as the target words to assess learning following the MALL-application intervention. Recommendations to follow best practice guidelines when it comes to addressing missing outcome data, clearly explaining statistical analyses, and considering a priori power analyses are also encouraged in order to improve methodological rigor and increase the replicability and reproducibility of the benefits of MALL-application on learning.

## 5. Conclusion and future directions

This meta-analysis identified 23 studies which systematically assessed L2 learning achievement outcomes by means of a MALL-application intervention in comparison to a traditional pen-paper learning control group. Based on these studies, we found a moderate-to-strong benefit of *g* = 0.88 of using MALL-application on L2 learning achievement over traditional classroom approaches, as well as across a wide range of moderator variables. This finding suggests that MALL-applications themselves are an effective way to boost L2 learning, and such experimentally validated MALL-applications should be considered for L2 learning. All included studies showed evidence of implementing learning principles in their MALL-applications, however none manipulated or compared these principles directly and the overall sample size of the included papers is small. Therefore, it is hard to determine their individual contribution to learning. Future studies should be dedicated to examining which pedagogical learning principles are most conducive for L2 achievement in a MALL-application setting. The beneficial effects of MALL-application on learning are not, however, without risks. Limitations revealed in the current work were mainly related to short intervention durations, missing follow-up measurements after the actual intervention, lack of randomization, and unclear measurement of the outcome variable. Such risks should motivate future research to utilize best research practices in order to produce replicable and valid effects. Taken together, MALL-application appear to be beneficial for L2 learning achievement, however the low number of studies in combination with the observed risks and limitations and the missing manipulation of learning principles require further research efforts to determine the impact of MALL-application in educational contexts and memory.

## Data Accessibility Statement

The data and methods reported here are available at the Open Science Framework: https://osf.io/htybd/.

## Additional File

The additional file for this article can be found as follows:

10.5334/pb.1146.s1 Supplementary Material. Criteria Definitions for Subgroup Analysis.
