# Supplementary material for: A Meta-Analysis on Mobile-Assisted Language Learning Applications: Benefits and Risks
Source: Psychol Belg. 2022 Sep 16;62(1):252–71. doi: 10.5334/pb.1146 (PMC9479751; doi:10.5334/pb.1146)
Supplement: Supplementary Material. — Criteria Definitions for Subgroup Analysis. [file pb-62-1-1146-s1.pdf]

Supplementary Materials for

A meta-analysis on mobile-assisted language learning applications: Benefits and risks

Mariela Mihaylova[1,2], Simon Gorin[1], Thomas P. Reber[1], Nicolas Rothen[1]

[1] Faculty of Psychology, UniDistance Suisse

[2] Department of Psychology and Educational Sciences, University of Geneva

This PDF file includes:
Figure S1
Criteria Definitions for Subgroup Analysis
Tables S1-S2

Figure S1

Detected outliers in meta-analysis

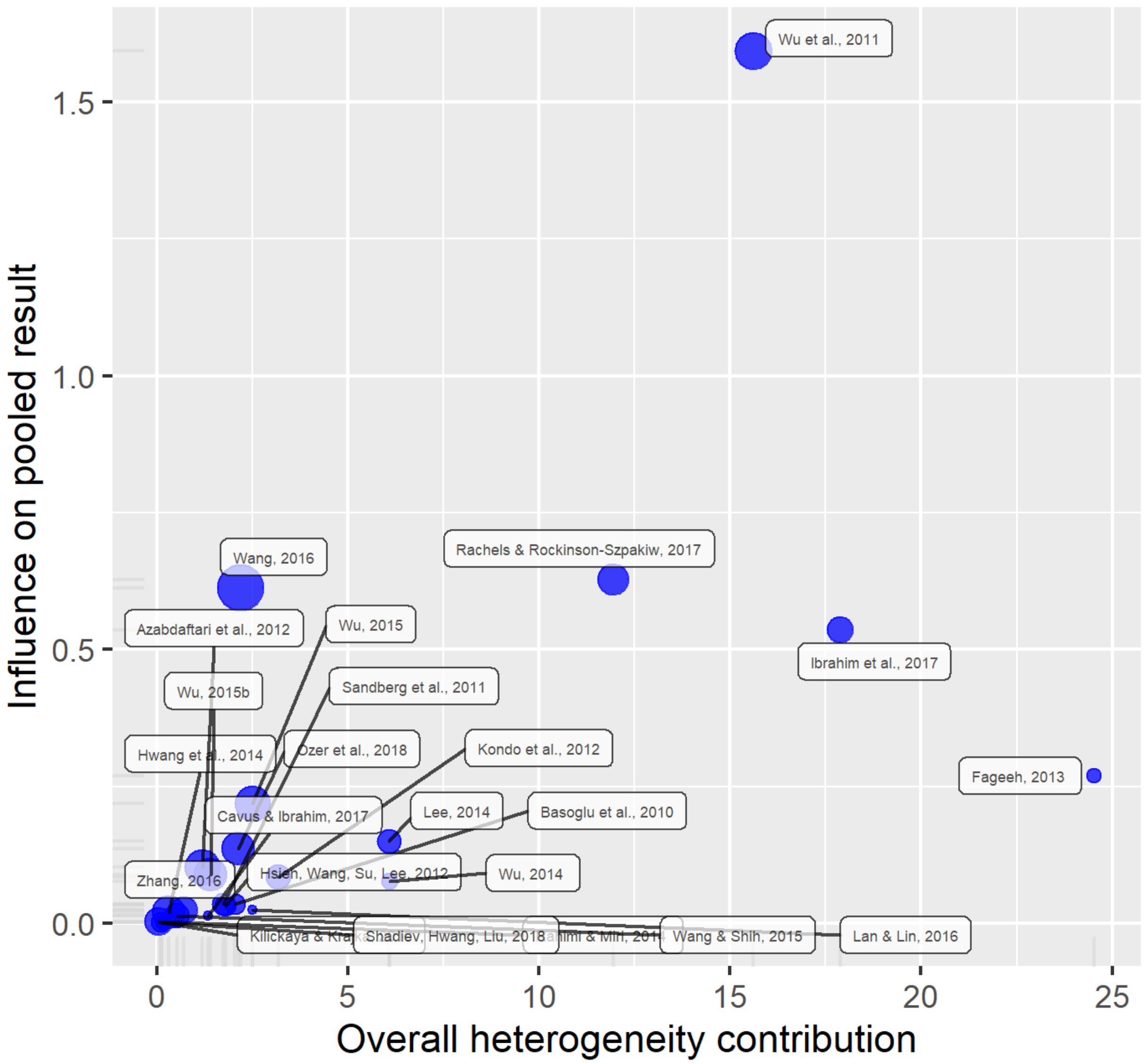

Note: Results of outlier analysis showing articles A2, A7, A13, and A20 as strong contributors to heterogeneity (*x*-axis) and influencing the overall pooled result (*y*-axis). The majority of the studies are clustered in the lower left corner, indicating lower heterogeneity contribution.

Criteria Definitions for Subgroup Analysis

School level: To be included in this subgroup, the paper should explicitly state that the age and school level of the participants. If only “university students” is mentioned, then classify into university level. If only ages are mentioned but no school level, we designated 18 years as the cut-off age for inclusion in university learners group; younger ages in the young learners group. Younger learners were further divided into secondary or high school (grades 9-12), middle school (grades 6-9) and primary school (grades 1-5).

Learning focus: Depends on the focus of the task performed with the application in the paper. If the focus was word learning such as with flashcards or mobile dictionary, it is primarily vocabulary unless otherwise mentioned in the paper. If performed a variety of learning topics, such as grammar, writing, reading, classify into other which includes multiple learning areas.

Type of app: Unless otherwise stated in the paper (such as when authors state they have created or developed the application or learning system used in that study), safe to assume the application exists already.

Duration of intervention: Classify based on what explicitly mentioned in article regarding how long participants used the app to learn before the post-test. If exact weeks not specified and only a semester is specified, assume 15 weeks per semester. If participants could use the device in their free time in addition to intervention, classify based on controlled intervention portion of study only for which duration info available.

Learning principles: inclusion/exclusion criteria listed in Table S1. Efforts should be made to stick to what is explicitly described in the paper.

Table S1

Classification criteria for learning principles

Learning Principle	Inclusion	Exclusion
Retrieval	Participants were prompted to actively recall study material, also during learning phase	Materials were re-read or re-studied passively or not explicitly mentioned in article, multiple choice test performed
Distributed Learning	Learning/intervention took place over time, several different sessions	Intervention/learning took place during one session
Feedback	Feedback was given to participants directly in the application	No feedback given to participants during learning/intervention or not explicitly mentioned in article
Multisensory Learning	Learning material was presented in multiple modes during intervention (i.e – audio and visual)	Learning material was presented in one method only (i.e –text only)

Note: The inclusion criteria raters followed when rating each article for learning principles

Table S2

Inter-Rater Reliability Scores

Subgroup	Fleiss Kappa before discussion
Learning Stage	0.95
Learning Focus	1.00
Learning Mode	0.95
Duration	1.00
Learning Principles – RP	0.70
Learning Principles – FB	0.88
Learning Principles – MM	0.93

Note. Fleiss Kappa values before rater deliberation. RP = retrieval practice, FB = feedback, MM = multimodal.
